# Water, sanitation, and hygiene (WASH) analysis of Amansea and Ugwuoba livestock market settlements and bacteriological quality investigation of drinking water sources at Amansea, Nigeria

**DOI:** 10.4314/ahs.v25i4.4

**Published:** 2025-12

**Authors:** Gilbert Karngong Nfor, Vincent Nnamdigadi Chigor, Lewis Iheanacho Ezeogu, Cornelius Arome Omatola

**Affiliations:** 1 Water and Public Health Research Group, Department of Microbiology, University of Nigeria, Nsukka, Nigeria; 2 Centre for Environmental Management and Control, University of Nigeria, Enugu Campus, Nigeria; 3 Department of Microbiology, Faculty of Biological Sciences, University of Nigeria, Nsukka, Nigeria; 4 School of Health Sciences, Catholic University of Central Africa; 5 Department of Microbiology, P.M.B. 1008, Kogi State University, Anyigba, Nigeria; 6 Institute for Water and Wastewater Technology, Durban University of Technology, P.O. Box 1334, Durban 4000, South Africa

**Keywords:** Water, sanitation, hygiene, faecal coliform, total coliform

## Abstract

**Background:**

Water, sanitation, and hygiene (WASH) are 3 interconnected drivers of waterborne diseases. This study investigated the WASH status of Amansea and Ugwuoba Livestock Market Settlements (LMSs).

**Methods:**

Data on drinking water sources, sanitation and hygiene practices were obtained from randomly selected households in Amansea (140) and Ugwuoba (248) LMSs. In addition, the drinking water samples (borehole and commercial sachet water) were collected monthly (September 2020 - August 2021) from the LMSs for bacteriological analysis.

**Results:**

Of the 388 households surveyed, 195 (50%) had access to improved drinking water sources in both LMSs. The households in Amnsea had more of improved drinking water sources compared to those in Ugwuoba (67.1%; 94/140 vs 40.7%; 101/248). Most households in Amansea (64.3%; 90/140) and Ugwuoba (58.5%; 145/248) LMSs practiced open defecation. Large proportions of households in Amansea (75.3%, 61/81) and Ugwuoba (55.0%, 77/140) had inadequate hygiene facilities. The borehole samples had a mean total and faecal coliform counts of 26 CFU/100 mL and 35 CFU/100 mL, respectively, which was undetected in the commercial sachet water.

**Conclusion:**

Fewer households in Ugwuoba than Amansea had access to improved drinking water. Most of the households in both LMSs do not have access to improved sanitation, and sanitation facilities. The faecal pollution of the borehole water sources suggests the needs for treatment of drinking water to prevent any possible waterborne outbreak in the area. Further, increased access to improved drinking water, sanitation, and sanitation facilities in both LMSs should be promoted by the relevant government agency.

## Introduction

Safe drinking water, sanitation and hygiene (WASH) play essential role to human health and general well-being. In addition, it contributes significantly to environmental protection, better educational outcomes, prevent chain of disease transmission and help promote the overall resilience of communities to climate shocks^1^. Water, as a critical component of WASH, serves a variety of activities, including nutrient delivery, waste elimination, temperature regulation, lubrication, and structural support. Thus, limited access to safe water, sanitation, and hygiene (WASH) has severe repercussions for public health, particularly among children, who may be more prone to infection and have a greater risk of illness due to their lower immunity^2^.

Water becomes unsafe when contaminated with faecal matter. Domestic sources of faecal contamination of water include wastewater from toilets and bathrooms. In addition, faecal matter from animal sources could equally contribute to aquatic faecal deposits^3^,^4^,^5^. The faecal matter from animal sources could include ranches, normadic herding or livestock markets. Thus, the bacteriological quality of drinking water is often assessed by enumerating Faecal Indicator Bacteria (FIB) such as faecal coliforms, total coliforms, enterococci, and faecal streptococci on selective agars and comparing the counts against international (such as World Health Organisation (WHO) and national standard guidelines (such as the Nigerian Standards for Drinking Water Guidelines (NSDWQ) for compliance. The presence of FIB in water suggests the presence of other pathogens which is a pointer to an infection risk following contact with the water^6^.

Infectious illnesses are largely spread via hands. Although regular handwashing with soap and water is a very efficient way to keep people healthy, yet a large number of people lack access to facilities for handwashing with water and soap^3^. According to World Bank, hygiene promotion is the most cost-effective health intervention^3^. Unhygienic water collection, storage, and lack of home water treatment methods do lead to the spread of diseases and bacterial contamination^4^. In order to provide a sustainable WASH intervention in communities, knowledge, attitudes, and practices (KAP) in these areas are essential. Lack of WASH awareness and WASH practices are usually important contributors to the incidence of water-borne diseases in communities such as Livestock Market Settlements (LMSs)..^7^ WASH-related health consequences have been demonstrated to be directly positively correlated with WASH knowledge, practices, and behaviours, where humans are ignorant of the health importance of quality drinking water, proper sanitation, and hygienic practices^4^. Children's faeces that are believed to be harmless in homes, open defecation into water bodies and near houses in communities, and poor sanitation are among the factors that have raised the risk of disease transmissions, particularly to children via faecal-oral routes^4^.

Globally, 2.2 billion (1 out of every 3 persons) do not have safe drinking water; 4.2 billion people lack safe sanitation, and 3 billion people lack basic handwashing facilities at home^3^. Over 1.8 billion people in the world use a source of drinking water that is faecally contaminated placing people at risk of contracting diseases such as cholera, dysentery, typhoid, and polio^3^. According to a WHO report, improving access to potable water and improving environmental conditions might avert 94 % of all diarrheal infections^2^. A recent WASH-associated burden of disease estimates showed that approximately 1.4 million people die annually due to inadequate drinking water, sanitation and hygiene. The death rates due to inadequate WASH were higher in low and middle income countries compared to the developed countries. Of the death rates, unsafe sanitation alone accounted for 564,000, mainly from gastroenteritis related complications^8^. Out of a population of 223,804,632 Nigerians, only about 29.0 % and 32.0 % use safely managed drinking water and sanitation services, respectively^9^. A safely managed drinking water source is an improved source that is accessible on premises, available when needed, and uncontaminated by hazardous chemicals and faeces^3^. A safely managed sanitation facility is an improved sanitary facility where faeces are treated off-site or safely disposed of in situ, and which is not shared with other households^9^. In the country, water-borne illnesses are responsible for approximately 130,000 child fatalities each year^2^.

The demand for livestock products in Nigeria has increased remarkably due to population and income growth, as well as urbanization. This has resulted in the widespread distribution of market settlements in Nigeria such as the Amansea and Ugwuoba Livestock Market Settlements (LMSs) situated in Anambra and Enugu state, Nigeria respectively. Most of the LMSs are situated at urban peripheries were accessibility to good quality water is often challenging. In addition LMSs are usually associated with temporary housing structures which lack good sanitation facilities resulting to an increase in the practice of open defecation. The need for sustainable and improved WASH conditions among LMSs in Nigeria is therefore imperative for improved public health. Also, there is a dearth of data on WASH and water quality related data among LMSs in Nigeria. Therefore, the current study assessed the WASH status of Amansea and Ugwuoba LMSs and further investigated the bacteriological quality of drinking water sources at the Amansea LMS.

## Methodology

### Study area

This research was carried out at Amansea and Ugwuoba in Anambra and Enugu states of Nigeria, respectively. Amansea is situated in the Awka-North Local Government Area of Anambra State. Awka-North Local Government Area is found in the tropical rainforest region and is situated between latitude 6°11′ - 6°17′ N and longitude 7°02′ E - 7°08′ E. It has a humid climate and an average daily relative humidity of 79.4%. Its annual rainfall ranges from 2000 mm to 3000 mm and the mean daily maximum and minimum air temperatures are 32.2°C and 23.3°C, respectively. Awka region consists of low-lying fertile land plains. Its inhabitants are mostly civil servants, craftsmen, farmers, and traders^10^. Awka has a projected population of about 4 million^11^. Drinking water sources at Amansea include boreholes, wells, and sachet water. Ugwuoba is situated on top of a hill in Nigeria's rainforest region. The average annual rainfall in the area is 1450mm, with 80% relative humidity. The average annual temperature ranges from 22 to 34 °C. Ugwuoba is located in Enugu State's Oji River Local Government Area^10^. Amansea and Ugwuoba are villages in Anambra and Enugu states respectively. The LMSs in these two villages are communities of Fulanis and Hausas (mainly) and their families who are not indigenous to these villages but settle within the villages mentioned for the chief purpose of their livestock market activities

### Water sample collection

A total of 36 drinking water samples were collected/purchased monthly for analysis over 12 months stemming from September 2020 to August 2021. Midstream borehole water (totaling 12 samples) flowing from a tap was collected in the morning into a wide-mouthed 500 mL sterilized glass bottle. The purpose of a midstream water collection was to limit the possibility of contamination of the borehole water flowing through the tap when the tap was opened. In this technique, water was first allowed to flow for about 2 mins. The water was then collected into the collection bottle while the tap was still flowing. Sachet water (totaling 24 samples: 12 from each brand) from two different brands was purchased from randomly selected stores in the Amansea LMS. The bottles were filled leaving a top space of 2.5 cm to enable easy shaking. Samples, collected/purchased were transported on ice to the laboratory and processed within 8 hours of sampling.

### Sample size determination

The formula below was used in computing the sample size for the participants recruited in the WASH study:


$$N=\frac{Z^{2}P(1-P)}{\delta^{2}}$$

The sample size was determined using P of 45%, the reported percentage of households with safe drinking water in a related study in Zimbabwe carried out by Makokove et al^12^. Where N represents the sample size, Z is the standard score corresponding to 95% confidence level and a margin of error/of the study of 5%. This gives a sample size of 380 participants. A non-response rate of 2% was added to the sample size, giving a total sample size of 388 participants. This was distributed across the two LMSs: Amansea LMS (140) and Ugwuoba LMS (248). The discrepancy is mainly due to size. Ugwuoba is a much larger LMS compared to Amansea LMS

### Survey data collection

This study was based on a survey design. The multiple indicator cluster survey (MICS)13 was used and a total of 388 adapted questionnaires were used to generate data regarding drinking water sources and sanitation and hygiene practices from randomly selected households in the Amansea (140) and Ugwuoba (248) LMS. The MICS is a household survey program that gathers information on range of subjects such as WASH, to assist nations in tracking the welfare of their citizens, particularly women and children. A parent from each of the households who agreed to participate in the survey was interviewed regarding the drinking water sources, treatment and storage for the water analysis we sought. For the sanitation analysis, key questions regarding type of sanitation facility, sharing of sanitation facility and infant sanitation was asked. Lastly, key questions regarding handwashing practices were asked for the hygiene analysis. We sought permission to observe the household's main handwashing station and drinking water storage containers for on-the-spot observations, and when granted, we visually assessed for the presence of soap, detergent, or other cleaning products andthe state of the drinking water storage container respectively. In addition, we sought and obtained administrative clearance before commencement of study among LMS settlers. The operational definitions are included in the supplementary file.

### Inclusion and exclusion criteria

Prior to the WASH survey, an informed consent form was given to each participant. Only those who agreed to participate in the survey and provided written/oral consent were recruited in the study. In addition, only those living in each of the LMSs were allowed to participate in the study. Furthermore, study was limited to only household members aged 18 and above. Individuals who were under 18 years, did not agree to participate in the study and were not living in each of the two LMSs were excluded from participation in the study

### Bacteriological analysis of water samples

Bacteriological (faecal coliform count and total coliform count) analysis was carried out using the method described^14^. First, tenfold serial dilution of samples and inoculation of media plates were carried out under standard microbiological techniques. For each water sample collected, 100 mL of an appropriate dilution of the sample predetermined from a pilot study was filtered through a membrane filter paper (diameter, 25 mm; pore size, 0.45 µm) (Meck, Germany) using a membrane filtration pump (Farntec, China). The filter paper containing the trapped bacteria was then inoculated on the prepared mEndo and mFC agar plates. mEndo and mFC were formulated based on the manufacturer's instructions. The mEndo plates were incubated at 37 °C for 24 hours while the mFC plates were incubated at 44.5 °C for 24 hours. After incubation, blue colonies on mFC agar plates and green metallic sheen colonies on mEndo plates characteristic of faecal coliforms (FC) and total coliforms (TC) respectively were counted and recorded in CFU/100 mL. Analysis was done in triplicates.

### Data analysis

The statistical packages for social sciences (SPSS) version 21 was used to analyze the data obtained in this study. The chi-square test or one-way ANOVA was used where appropriate to compute p-values. Probability values of <0.05 were considered significant.

## Results

### Household demographics

The survey revealed that the majority of the respondents in this study were males in both Amansea LMS (103/140; 73.6%) and Ugwuoba LMS (186/248; 75.0%) with Ugwuoba LMS recording a higher percentage of males. The father was the breadwinner in 68.6% (96/140) and 79.0% (198/248) of the households surveyed in Amansea LMS and Ugwuoba LMS, respectively. Based on the educational status of respondents, 27.9% (39/140) and 27.4% (68/248) of respondents in Amansea LMS and Ugwuoba LMS respectively have no formal education. The highest form of education amongst the respondents in both LMS was primary education. Overall, Amansea LMS recorded a lower rate of education (34.3%, (48/140) while Ugwuoba LMS recorded a higher rate of education [44.8%, (111/248)]. The second highest form of education amongst the respondents in both LMS was secondary education. In this light, Amansea LMS recorded a higher rate of educated respondents (31.4%, (44/140) than Ugwuoba LMS [18.9%, (47/248)]. The majority of respondents in this study were within the average total monthly income bracket of ₦ 20000- ₦ 29000 for both local market settlements with Amansea LMS recording 25% (35/140) and Ugwuoba LMS recording 23.8% (59/248). The predominant age bracket of respondents in this study was 36-45 years for both LMS with Amansea LMS recording 35.7 % (50/140) and Ugwuoba LMS recording 31.9 % (79/248). This was followed by the age bracket, 26-35 years with Amansea LMS recording 33.6% (47/140) and Ugwuoba LMS recording 27.0% (67/248). The majority of the respondents in this study were the heads of their households in Amansea LMS (79.3%; 111/140) and Ugwuoba LMS (71.4 %; 177/248). Among the respondents who were not the heads of their households, the majority were wives to the heads of the households. In this light, Amansea LMS recorded 41.4 % (12/29) while Ugwuoba LMS recorded 57.7% (41/70). Table 1 shows the descriptive household characteristics of the survey respondents.

**Table 1:** Descriptive statistics of household characteristics of Amansea and Ugwuoba LMS

Household characteristics		Amansea LMS		Ugwuoba LMS	

		Frequency	%	Frequency	%
Gender of the respondent	Male	103	73.6	186	75.0
	Female	37	26.4	62	25.0
	Total	140	100.0	248	100.0
Breadwinner of household	Father	96	68.6	198	79.0
	Mother	10	7.1	13	5.2
	Child	2	1.4	0	0.0
	Self	31	22.1	27	10.9
	Brother	1	0.7	12	4.8
	Total	140	100.0	248	100.0
The highest educational level attained by the breadwinner of the household	None	39	27.9	68	27.4
	Informal education	7	5.0	9	3.6
	Primary education	48	34.3	111	44.8
	Secondary education	44	31.4	47	18.9
	University	0	0.0	1	0.0
	Do not know	2	1.4	4	1.6
	Total	140	100.0	248	100.0
Average total monthly income for the household (naira/month)	<10000	14	10.0	22	8.9
	10000-19000	20	14.3	31	12.5
	20000-29000	35	25.0	59	23.8
	30000-39000	23	16.4	52	21.0
	>40000	9	6.4	21	8.5
	Do not know	39	27.9	63	25.4
	Total	140	100.0	248	100.0
Age of respondent	18-25	21	15.0	56	22.6
	26-35	47	33.6	67	27.0
	36-45	50	35.7	79	31.9
	46-55	16	11.4	25	10.1
	>55	3	2.1	7	2.8
	Refused to answer	3	2.1	14	5.6
	Total	140	100.0	248	100.0
Respondent is the head of the household	Yes	111	79.3	177	71.4
	No	29	20.7	71	28.6
	Total	140	100.0	248	100.0
Relationship of the respondent with the head of household in the case where he/she is not the head of the household	Wife	12	41.4	41	57.7
	Mother	3	10.3	7	9.9
	Daughter	4	13.8	1	1.4
	Father	0	0.0	2	2.8
	Son	10	7.1	7	9.9
	Brother	0	0.0	13	18.3
	Total	29	100.0	71	100.0

### Water supply, treatment, storage, continuity, sourcing and use

#### Drinking water supply

**Figure 1:**
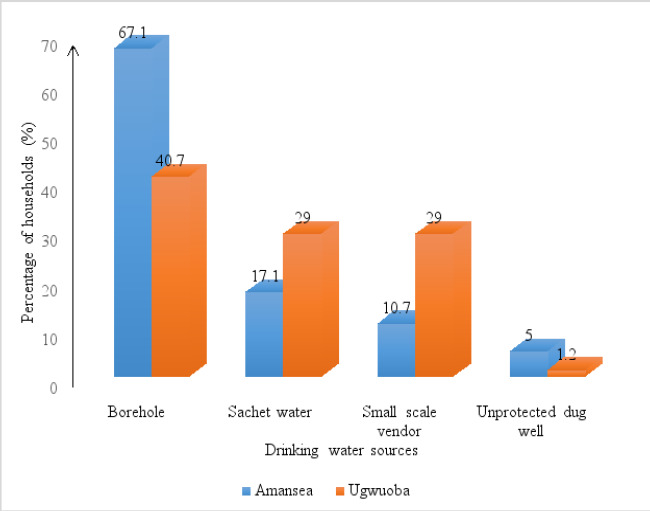
Percentage of households obtaining drinking water from the various water sources at Amansea and Ugwuoba livestock market settlements

Borehole water was the predominant drinking water source among households in Amansea (67.1%; 94/140) and Ugwuoba (40.7%; 101/248) LMS while the drinking water source least used was unprotected dug well in Amansea (5.0%; 7/140) and Ugwuoba (1.2%; 3/248) LMS (p<0.05). Figure 1 shows the water source distribution across the two LMS.

#### Water treatment

**Figure 2:**
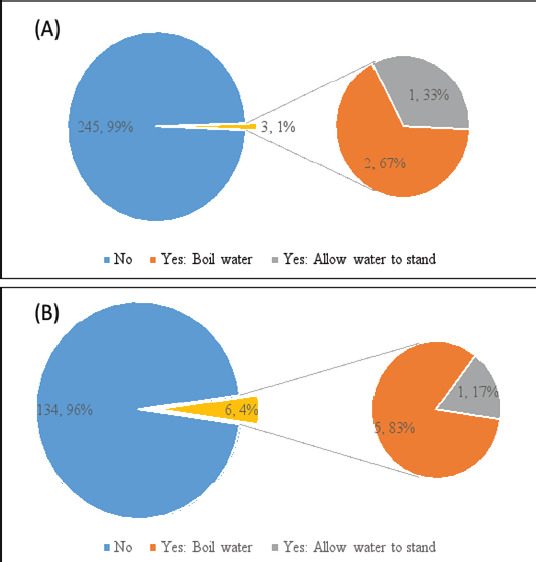
Percentage of households at Amansea (A) and Ugwuoba (B) who treat water before drinking. Treatment methods, with the percentage of households employing these, are shown in the expanded pie chart

The data collected in this survey revealed that only 4.3% (6/140) and 1.2% (3/248) of the households at Aman-sea and Ugwuoba respectively treat their drinking water. Among those that treat water, boiling water was the most used water treatment method with Amansea recording 83.3% (5/6) and Ugwuoba recording 66.7% (2/3). Figure 2 shows the percentage of households at Amansea and Ugwuoba LMS who treat water before drinking and the treatment methods used.

#### Water Storage

Water storage among the households in this study area was also assessed and it was noted that 70.7% (99/140) and 64.1% (159/248) of households in Amansea LMS and Ugwuoba LMS respectively acknowledged that they have a separate drinking water storage container in their home. Out of the number of households that have a separate drinking water storage container in both LMSs, 86.9% (86/99), 7.1% (7/99), and 6.1% (6/99) households had plastic, metal, and earthen storage containers respectively for Amansea LMS while 91.2% (145/159), 1.9% (3/159) and 6.9% (11/159) had plastic, metal, and earthen storage containers, respectively for Ugwuoba LMS. The most used method of removal of water from drinking water storage containers across both LMSs was the use of a cup. In this light, Amansea LMS recorded 77.8% (77/99) while Ugwuoba recorded 59.1% (94/159). The frequency of cleaning drinking water storage containers among households having drinking water storage containers in this study site was also investigated. It was discovered that the majority of these households clean their drinking water storage containers daily with Amansea LMS and Ugwuoba LMS recording 55.6% (55/59) and 67.9% (108/159), respectively. Out of all respondents in this study area who acknowledged the availability of drinking water storage containers in their households, 78.8% (78/99) and 100% (159/159) accepted to show their household's drinking water storage container in Amansea LMS and Ugwuoba LMS respectively. Among respondents who accepted to show the drinking water storage container of their households, the most predominant observations were: the presence of a separate (dedicated) cup for carrying out drinking water from the storage vessel in households of Amansea LMS (69.2%; 54/78) and Ugwuoba LMS (58.5%; 93/159); drinking water storage container is covered in households of Amansea LMS (84.6%; 66/78) and Ugwuoba LMS (84.3%; 134/159); drinking water storage container has a narrow neck in households of Amansea LMS (51.2%; 40/78) and Ugwuoba LMS (67.9%; 108/159); drinking water storage container looks clean in households of Amansea LMS (82.1%; 64/78) and Ugwuoba LMS (66.7%; 106/159). Table 2 shows the drinking water storage parameters assessed in this study and their proportions across the two LMS.

**Table 2:** Drinking water storage characteristics across Amansea and Ugwuoba LMS

Drinking water storage characteristics	Categories	Sub-categories	Amansea LMS	Ugwuoba LMS

			Frequency (%)	Frequency (%)
Have separate drinking storage container	Yes		99 (70.7)	159 (64.1)
	No		41 (29.3)	89 (35.9)
	Total		140 (100.0)	248 (100.0)
Material type of drinking water storage container	Plastic		86 (86.9)	145 (91.2)
	Metal		7 (7.1)	3 (1.9)
	Earthen pot		6 (6.1)	11 (6.9)
	Total		99 (100.0)	159 (100.0)
Method of removal of water from drinking water storage container	Tap		2 (2.0)	5 (3.1)
	Cup		77 (77.8)	941 (59.1)
	Pour from container		20 (20.2)	60 (37.7)
	Total		99 (100.0)	159 (100.0)
Frequency of cleaning drinking water storage container	Daily		55 (55.6)	108 (67.9)
	Severol times per weak		5 (5.1)	6 (3.8)
	Once a week		22 (22.2)	9 (5.7)
	Once a month		4 (4.0)	7 (4.4)
	Do not know		13 (13.1)	29 (18.2)
	Total		99 (100.0)	159 (100.0)
Respondent shows drinking water storage container	Yes		78 (78.8)	159 (100.0)
	No		21 (22.2)	0 (0.0)
	Total		99 (100.0)	159 (100.0)
Observation about drinking water storage container	Cup off the floor	Yes	54 (69.2)	93
		No	5 (6.4)	8
		Not applicable	19 (24.4)	58
		Total	78 (100.0)	159.0
	Drinking water storage container covered	Yes	66 (84.6)	134 (84.3)
		No	11 (14.1)	25 (15.7)
		Not applicable	0 (0.0)	0 (0.0)
		Total	78 (100.0)	159 (100.0)
	Drinking water storage container has a narrow neck	Yes	40 (51.2)	108 (67.9)
		No	38 (48.7)	51 (32.1)
		Not applicable	0 (0.0)	0 (0.0)
		Total	78 (100.0)	159 (100.0)
	Drinking water storage container looks clean	Yes	64 (82.1)	106 (66.7)
		No	14 (17.9)	53 (33.3)
		Not applicable	0 (0.0)	0 (0.0)
		Total	78 (100.0)	159 (100.0)

LMS=Livestock Market settlement

#### Water sourcing

##### Continuity of water supply

Within the last 6 months to the time this survey was conducted, 97.1% (136/140) and 100% (all respondents) at Amansea LMS and Ugwuoba LMS respectively indicated that they experienced no interruptions in their drinking water supply from the main drinking water source (Table 3).

**Table 3:** Water supply management at Amansea LMS and Ugwuoba LMS

Water parameters		Amansea LMS		Ugwuoba LMS	

		Frequency	Perc (%)	Frequency	Perc (%)
MS of water for domestic use (washing of clothes, dishes, bathing)	Borehole	119	85.0	189	76.2
	Unprotected dug well	2	1.4	2	0.8
	Small scale vendor	7	5.0	46	18.5
	River	12	8.6	11	4.4
	Total	140	100.0	248	100.0
Size of container used for fetching water (litres)	20	23	16.4	114	46
	25	108	77.1	134	54.0
	50	9	6.4	0	0.0
	Total	140	100.0	248	100.0
Use of transportation means to fetch water	Yes	28	20.0	9	3.6
	No	112	80.0	239	96.4
	Total	140	100.0	248	100.0
Transportation means for fetching water	Motor cycle	3	10.3	3	33.3
	Wheelbarrow	25	86.2	6	66.7
	Vehicle	1	3.5	0	0.0
	Total	29	100.0	9	100.0
Duration of one round trip of water collection (mins)	≤30	64	45.7	140	58.5
	31-60	4	2.9	13	5.2
	Do not know	42	30.0	23	9.3
	Not applicable	30	21.4	72	29.0
	Total	140	100.0	248	100.0
Experienced breaks in DWS from the MS in the last 6 months	Yes	4	2.9	0	0.0
	No	136	97.1	248	100.0
	Total	140	100.0	248	100.0
Showed how or where household disposes used water	Yes	92	65.7	194	78.2
	No	48	34.3	54	21.8
	Total	140	100.0	248	100.0
Observation on points of discharge of household's used water	Soak-away	4	4.3	6	3.1
	Sanitation facility	2	1.4	66	34
	Open channel	17	18.5	15	7.7
	Street surface	17	18.5	26	13.4
	Space outside premises	46	32.9	76	39.2
	Premise's yard or garden	6	4.3	5	2.6
	Total	92	100.0	194	100.0
Observations about points of discharge of used water	Stagnant water pool	11	12.0	19	9.8
	Swampy area	15	16.3	14	7.2
	Lots of insects (FMB)	17	18.5	50	25.8
	Bad smell	62	67.4	148	76.3
	Signs of residues	18	19.6	69	35.6
	None	20	21.7	14	7.2

MS: Main source; DWS: Drinking water supply; FMMB: Flies, mo squitoes breeding; MSDW: Main source of drinking water

#### Duration of one round trip of water collection

The majority of respondents in Amansea LMS and Ugwuoba LMS use 30 minutes or less forone round trip of drinking water collection including queuing time. In this light, 45.7% (64/140) and 56.5% (140/248) were recorded for Amansea LMS and Ugwuoba LMS respectively (Table 3). The “not applicable category” was assigned to respondents whose water source originated from a small-scale water vendor (Table 3).

#### Water use

Apart from drinking, the households in this study use water for several domestic purposes including washing clothes, washing kitchen utensils, and/or bathing. Table 3 shows the main sources of water for domestic purposes. The borehole was the main water source used by the household for domestic purposes across both LMS with occurrence rates of 85% (119/140) and 76.2% (189/248) of households at Amansea LMS and Ugwuoba LMS respectively. Out of the total number of respondents in this study, 65.7% (92/140) and 78.2% (194/248) from Amansea LMS and Ugwuoba LMS, respectively accepted to show how or where households dispose used water. It was discovered that the majority of households in both LMS (who agreed to show where or how used water is disposed of) use the space outside the premises for the disposal of used water. In this light, Amansea LMS recorded 32.9% (46/92) and Ugwuoba recorded 39.2% (76/194). Across both LMS, the highest observation about the point of discharge of used water was bad smell with Amansea recording 67.4% (62/140) and Ugwuoba recording 76.3% (148/248).

#### Classification of Water sources based on WHO/UNICEF JMP (Joint Monitoring Program) ladders Improved and Unimproved drinking-water sources

**Figure 3:**
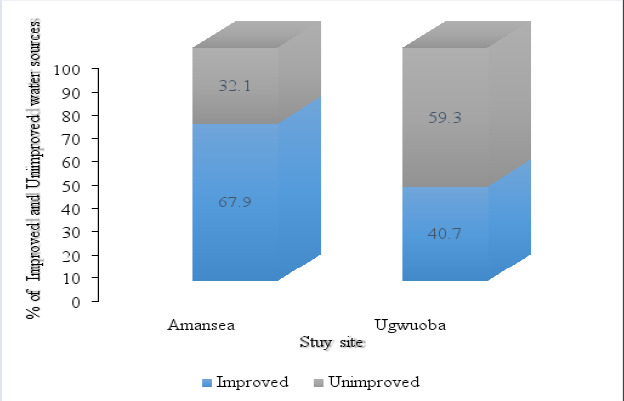
Drinking water sources (improved and unimproved) in households in Amansea and Ugwuoba LMS

Households in Amansea LMS recorded a higher proportion of improved drinking water sources, 67.9% (95/140) than the households at Ugwuoba LMS which had a much lower proportion of improved water sources, 40.7% (101/248) (Figure 3).

#### Basic and limited drinking-water sources

**Figure 4:**
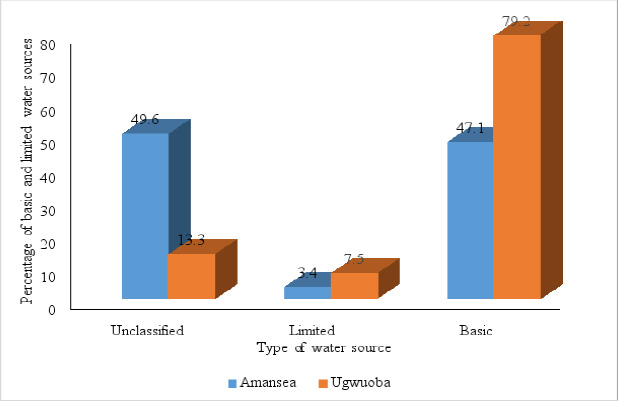
Drinking water sources (basic and limited) in households in Amansea and Ugwuoba LMS

The improved water sources in this study were further grouped into basic, limited, and unclassified. The unclassified category was assigned to respondents who did not know the duration of one round trip of drinking water collection from the water source including queuing or for respondents whose water source originated from a small-scale water vendor. Ugwuoba LMS recorded a higher proportion of basic water sources (68.3%, 69/101) than Amansea LMS (48.4%, 46/95) (Figure 4).

#### Hygiene

##### Hygiene practice

About 58% (81/140) of respondents of Amansea and 57% (140/248) of Ugwuoba LMSs agreed to show how or where household members wash their hands. Of the respondents, 3.7% (3/81) and 35.7% (50/140) households in Amansea and Ugwuoba LMS, respectively neither had a handwashing facility nor water nor soap at the facility; 48.1% (39/81) and 42.9% (60/140) households in Amansea and Ugwuoba LMS, respectively had a handwashing facility without soap and water; 27.2% (22/81) and 12.1% (17/140) of households in Amansea and Ugwuoba LMS respectively had a handwashing facility with water but without soap and lastly 21.0% (17/81) and 9.3% (13/140) households in Amansea and Ugwuoba LMS, respectively had a handwashing facility with water and soap present at the facility. The data described above revealed that handwashing practices (although practiced by the minority) were more dominant amongst respondents in Amansea LMS than those in Ugwuoba LMS. However, 52.1% (73/140) and 56.0% (139/248) respondents in Amansea LMS and Ugwuoba LMS use soap for handwashing and or other purposes. Out of the respondents who had used soap for handwashing and or other purposes, 87.7% (64/73) and 90.7% (126/139) from Amansea LMS and Ugwuoba LMS, respectively responded that they used soap on the same day of or previous day to questionnaire administration. The most important purpose for using soap amongst respondents in both LMS who used soap on the same day of or the previous day of questionnaire administration was “washing body”. Respondents of Amansea LMS and Ugwuoba LMS recorded 96.9% (62/64) and 98.4% (124/126). The least purpose for using soap amongst respondents in both LMS who washed hands on the same day of or the previous day to questionnaire administration was “Washing hands before receiving visitors”. In this light, Amansea LMS and Ugwuoba LMS recorded 3.1% (2/64) and 2.4% (3/126). Quite fewer respondents amongst those in Amansea LMS who used soap on the day of or previous day to questionnaire administration used soap for hand-washing after defecating and before eating with records of 28.1% (18/64) and 39.1% (25/64) respectively. A similar pattern was observed for respondents in Ugwuoba LMS who wash hands with soap after defecating with records of 20.6% (26/126). The reported handwashing practices during the four critical moments (handwashing with soap after dfecating, before eating, before going out, and before receiving visitors) differed from the actual/observed practices. Table 4 gives more details on respondent's responses to questions regarding hygiene.

**Table 4:** Hygiene practices at Amansea and Ugwuoba livestock market settlements

Hygiene parameters		Amansea		Ugwuoba	

		Frequency	Perc (%)	Frequency	Perc (%)
Showed how (where) household members wash hands	Yes	81	57.9	140	56.5
	No	59	42.1	108	43.5
	Total	140	100.0	248	100.0
Hand washing facility with soap and water					
Facility (-), water (-) soap (-)	Yes	3	3.7	50	35.7
Facility (+), water (-) soap (-)	Yes	39	48.1	60	42.9
Facility (+), water (+) soap (-)	Yes	22	27.2	17	12.1
Facility (+), water (+) soap (+)	Yes	17	21.0	13	9.3
	Total	81	100.0	140	100.0
Have soap or something else used for hand washing and or other purposes	Yes, soap	73	52.1	139	56.0
	No	67	47.9	109	44
	Total	140	100.0	248	100.0
Used soap today or yesterday (USTY)	Yes	64	87.7	126	90.7
	No	9	12.3	13	9.3
	Total	73	100.0	139	100.0
USTY: Purpose	WC	^a^54	^b^84.4	^a^122	^b^96.8
	WB	^a^62	^b^96.9	^a^124	^b^98.4
	WHAD	^a^18	^b^28.1	^a^26	^b^20.6
	WHBE	^a^25	^b^39.1	^a^78	^b^61.9
	WHBG	^a^3	^b^4.7	^a^4	^b^3.2
	O				
	WHBR	^a^2	^b^3.1	^a^3	^b^2.4

WC: washing clothes. WB: washing body. WHAD: Washing hands after defecating. WHBE: washing hands before eating. WHBGO: washing hands before going out. WHBR: washing hands before receiving visitors.

a: frequency of respondents who used soap on the day or the previous day the research was conducted for the listed purposes (such as washing clothes, washing body, and so on) out of the total respondents who used soap for any purpose the day or previous day the research was conducted.

b: Percentages of the frequencies (a). + = presence and - = absence

##### Classification of hygiene parameters based on WHO/UNICEF JMP's ladders

**Figure 5:**
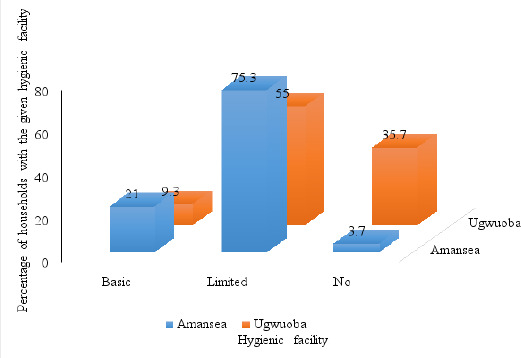
Hygiene facilities in households in Amansea and Ugwuoba LMS

Among the respondents who agreed to show their hygiene facilities (81/140 and 140/248 for Amansea and Ugwuoba LMS respectively), it was observed that a great proportion of respondents (35.7%; 50/140) in Ugwuoba LMS, as opposed to a minority (3.7%; 3/81) in Amansea LMS, had no hygiene facility; 75.3% (61/81) and 55.0% (77/140) of respondents in Amansea and Ugwuoba LMS respectively had limited hygiene facilities; 21.0% (17/81) and 9.3% (13/140) of respondents in Amansea and Ugwuoba LMS respectively had basic facilities (Figure 5).

#### Sanitation

##### Sanitation practice

The majority of respondents practice open defecation. Ninety out of 140 (64.3%) and 145/248 (58.5%) respondents in Amansea and Ugwuoba LMS respectively practice open defecation (Table 5). Faecal matter was deposited in the surroundings of 23.6% (33/140) and 21.8% (54/248) of respondents in Amansea LMS and Ugwuoba LMS respectively.

**Table 5:** Sanitation practice at Amansea and Ugwuoba

Sanitation parameters		Amansea		Ugwuoba	

		Frequency	Perc (%)	Frequency	Perc (%)
Kind of toilet used by the household	Open air/bush/field	90	64.3	145	58.5
	Bucket	0	0	9	3.6
	Pit latrine with slap	12	8.6	77	31.0
	Pit latrine without slap	8	5.7	16	6.5
	Flush to piped sewer system	5	3.6	0	0
	Flush/pour into the pit latrine	25	17.9	1	0.4
	Total	140	100.0	248	100.0
Kind of toilet based on sharing	Private (1 household)	33	66.0	85	90.4
	Shared (>1 household)	14	28.0	3	3.2
	Public	3	6.0	6	6.4
	Total	50	100.0	94	100.0
Respondent accepts to show the toilet	Yes	27	54.0	85	90.4
	No	23	46.0	9	9.6
	Total	50	100.0	94	100.0
Observation about toilet	VFR around the drop hole	9	33.3	20	21.3
	VFR on floor, wall, or door	4	14.8	8	8.5
	VUACM (tissue/paper)	1	3.7	3	3.2
	None	13	48.1	63	67.0
	Total	27	100.0	94	100.0
Observation about access to toilet	Path is clear	15	55.6	67	71.3
	Waste or debris on path	6	22.2	14	14.9
	Major crevice or pothole on path	2	7.4	6	6.4
	Mud on path	4	14.8	7	7.4
	Total	27	100.0	94	100.0
Kind of maintenance needed for household's toilet	Empty pit	1	2.0	2	2.1
	Dig new whole	2	4.0	0	0.0
	Cleaning	29	58.0	64	68.1
	None	18	36.0	28	29.8
	Total	50	100.0	94	100.0
The last time the youngest (<5yrs) child passed stool where did he/she defecate	Used the sanitation facility	2	5.4	1	2.0
	Used potty	13	35.1	11	22.0
	Used diapers	7	18.9	14	28.0
	Went in yard	4	10.8	9	18.0
	Went outside premises	11	29.7	15	30.0
	Total	37	100.0	50	100.0
The last time youngest (≤4yrs) child passed stool where were the faeces disposed of	Dropped into the toilet	3	8.1	5	10.0
	Washed away into the toilet	10	27.0	10	20.0
	Washed away, the water discharged outside	4	10.8	8	16.0
	Disposed of into the solid waste	3	8.1	1	2.0
	Disposed outside the yard	11	29.7	7	14.0
	Buried	2	5.4	16	32.0
	Did nothing	4	10.8	3	6.0
	Total	37	100.0	50	100.0
Feaces deposited in household surroundings	Yes	33	23.6	54	21.8
	No	107	76.4	194	78.2
	Total	140	100.0	248	100.0

VFR=Visible faeces residues; VUACM=Visible used anal cleansing material

As regards the location where the stool of under 5-year-olds was dropped after defecation, dropping of stool in to a sanitation (toilet) facility was the least recorded in Amansea (8.1%; 3/37) and Ugwuoba LMS (2.0%; 1/50).

##### Sanitation practice among children below 5 years

First, 26.4% (37/140) and 20.2% (50/248) of respondents in Amansea and Ugwuoba LMS respectively indicated the presence of children below 5 years in their households. The majority of respondents in Amansea LMS (35.1%; 13/37) reported that their children passed out stool in the potty when they defecated. However, the majority of respondents in Amansea LMS (29.7%; 11/37) who had children aged below 5 years reported that after their children defecated, the faeces were disposed of outside the yard. On the other hand, a majority of respondents in Ugwuoba LMS (30.0%; 15/50) reported that their under-5-year-old children went outside the premises to defecate. However, the majority of respondents in Ugwuoba LMS (32.0%; 16/50) who had children aged below 5 years reported that after their children defecated, the faeces was buried (Table 6). Use of sanitation (toilet) facility was the least recorded child defecation practice in Amansea (5.4%; 2/37) and Ugwuoba (2.0%; 1/50) LMS households.

##### Sharing of sanitation facilities

The study revealed that 66.0% (33/50), 28.0% (14/50), and 6.0% (3/50) of respondents in Amansea LMS who had toilet facilities (those not practicing open defecation) use a private toilet, shared toilet, and public toilet respectively. On the other hand, the study revealed that 90.4% (85/94), 3.2% (3/94), and 6.4% (6/94) of respondents in Ugwuoba LMS who had toilet facilities (those who did not practice open defecation) use a private toilet, shared toilet, and public toilet respectively, which were significantly different between the two LMS (p<0.05). The details of sanitation practices can be seen in Table 5.

#### Classification of sanitation facilities based on WHO/UNICEF JMP's ladders

##### Improved/Unimproved sanitation facilities

**Figure 6:**
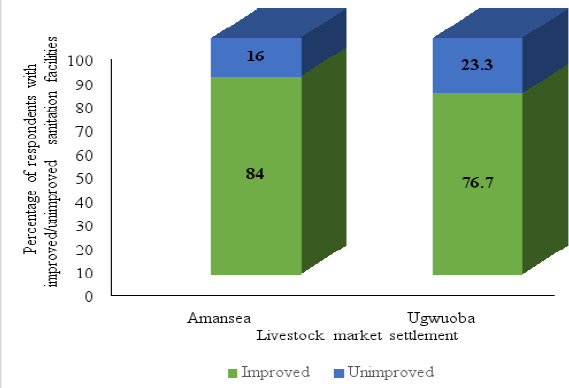
Households with improved/unimproved sanitation facilities based on JMP's ladders

This study showed that the majority of respondents who have toilets (don't practice open defecation) use improved sanitation facilities. In this light, Amansea LMS recorded 84.0% (42/50) while Ugwuoba LMS recorded 76.7% (72/94), however the difference in terms of the facilities used was not significant (p>0.05). Figure 6 shows the classification of sanitation facilities into improved and unimproved facilities.

##### Basic/Limited sanitation facilities

**Figure 7:**
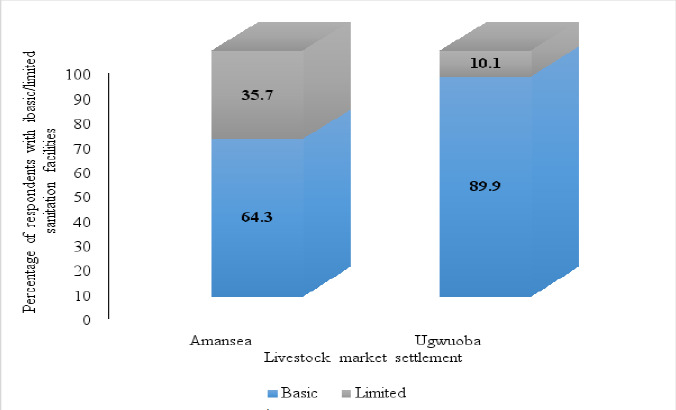
Households with basic/limited sanitation facilities based on JMP's ladders

The majority of the respondents with toilet facilities in Amansea and Ugwuoba LMS use basic sanitation facilities. This study revealed that 64.3% (27/42) and 35.7% (15/42) of respondents with toilet facilities in Amansea LMS use basic and limited facilities, respectively. On the other hand, 89.9% (71/79) and 10.1% (8/79) of respondents with toilet facilities in Ugwuoba LMS use basic and limited facilities, respectively and the difference was statistically significant (p<0.05) (Figure 7).

##### Bacteriological drinking water quality

**Figure 8:**
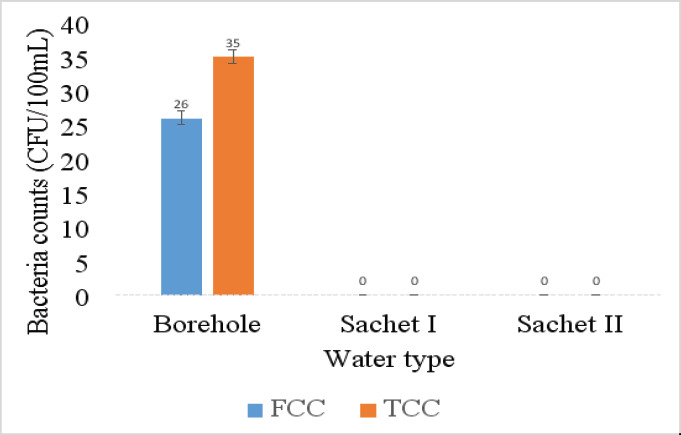
Variation in mean counts of faecal and total coliform bacteria of drinking water types at Amansea livestock market settlement. Samples were collected/purchased monthly at each site for a total of 12 months (September 2020 to August 2021) and each sample was analysed in triplicates. Reported values are the average counts for the entire 12-month period

Bacteriological water quality of the predominant drinking water types (borehole and the 2 sachet water brands) in Amansea LMS households was assessed. The results of the bacteriological assessment is presented in Figure 8. Borehole water recorded the highest total and faecal coliform counts with mean values of 35 CFU/100 ml and 26 CFU/100 ml, respectively while total and faecal coliform counts were undetected (0 CFU/100 ml) in both sachet water brands. One-way ANOVA revealed that there exists a significant difference in the total and faecal coliform levels across the drinking water types (p<0.05). Borehole water was noncompliant while both sachet brands were compliant to WHO and NSDWQ permissible limits for drinking water.

## Discussion

The lack of continuous and stable water supplies is a reality in many low-resource situations, forcing people to use a variety of water sources whose quality may be questionable. The gender characteristics of the respondents revealed that the majority were males across both LMSs. This could be due to the fact that livestock market activities are more predominant among males than females as shown by similar studies^15^,^16^. Also, the majority of those who engage in livestock farming activities are Fulani and Hausas who are predominantly Muslims. These individuals have been previously shown to believe (based on the Qur'an) that God has entrusted men the task of being responsible for women including breadwinning^17^. The majority of breadwinners in this study had a low level of education probably due to their inability to further sponsor their education post-primary levels as evident from their low monthly income levels of ₦ 20,000 – ₦ 29,000. This finding corroborates previous reports^18^ in Tanzania, which revealed that primary education was a more common educational status among livestock market farmers compared to non-livestock market farmers. The age 36-45 years predominance of respondents from both Amansea LMS and Ugwuoba LMS) implies the majority of the respondents were within the active working age brackets which is suitable for livestock market activities. This observation concurs with a similar study carried out elsewhere^19^ where livestock farmers aged 21-40 years with a mean age of 35.82 years predominated.

In this study, borehole water was the most common drinking water source compared to other water sources. On the contrary, another study has reported that river water was the most common water source among households in rural India^20^ a difference likely due to the inaccessibility and unavailability of borehole water in households of the latter study than the former. Generally, the current study observed a higher proportion of improved water sources in households in Amansea LMS compared to Ugwuoba LMS, an observation comparable with other reports elsewhere in the country^21^. One of the criteria for meeting the SDG target for safely managed drinking water is that the source be located on the premises. In terms of water accessibility, the survey found that none of the households in this study had their drinking water sources located on the premises, making accessibility difficult. In Amansea and Ugwuoba LMS, 45.7% and 58.5% of households respectively used ≤30 mins for one round trip of sample collection. This data shows that accessibility to a water source is poor and is contrary to the findings in Oforikrom^22^, Ghana recorded 65.0% and 60.0% of households having a drinking water source available on-premises and having to cover a distance of fewer than 30 mins round trip from their respective households to access their drinking water source respectively. This discrepancy may be due to the more advanced drinking water supply networks in Oforikrom, Ghana compared to that at Amansea and Ugwuoba LMS. The reason why the majority of the respondents used ≤30 mins for one round trip of drinking water collection could be due to the fact that Amansea and Ugwuoba are small livestock market settlements, hence water sources are not relatively far from households. Continuity of water supply (uninterrupted water supply) is as important as its availability. Studies have revealed that there is a correlation between the continuity of improved water sources and public health. One study^23^ discovered a relationship between disruptions in the piped water supply and suspected cholera infections in the city of Uvira in the Democratic Republic of Congo. Another study^24^ reported that increasing the piped supply to offer continuous service to 10% of the population of Hubli-Dharwad (a city in the state of Karnataka, India) was linked to a significant drop in typhoid among the poor in the beneficiary group. In this study, nearly all respondents at both LMSs indicated that they experienced no interruptions in the drinking water supply from the main drinking water sources in the last 6 months, suggesting that availability and accessibility to drinking water was not a problem to a majority of the households.

In the present study, very few households at both LMSs treat their drinking water, comparable with another household WASH-based survey reports of McGuinness et al.^20^ in rural India. The low water treatment practice rates in this study could be due to inadequate knowledge of water treatment practices. Among those that treat water, boiling water was the water treatment methods in both LMSs, similar to a previous report from a WASH-based study in Dhaka, Bangladesh^25^. The boiling method was used most in this survey probably because it is more cost-effective (especially for those living in rural areas where fuelwood is used at little or no cost in the boiling process) and an efficient method of destroying possible disease-causing microorganisms in the drinking water. Water treatment enhances the likelihood of having good-quality water in the home.

In the study, majority of households from both LMSs were found to store their drinking water in plastic containers which may play a multifaceted role in disease. First, most plastic-based storage containers contain bisphenol A, which can leach into water in significant amounts and cause health problems in children^21^. Secondly, the plastic storage containers have been shown to support the breeding of most species of the Anopheles mosquito in different geographical areas in Nigeria^26^,^27^. This study revealed a minority of households using earthen containers as their drinking water storage container type, which has been proven to be better due to the good supply of alkaline (clay) for maintaining optimum pH balance in the body^20^.

The use of a cup was identified as the most common method of drawing water from the drinking water storage container by households with separate drinking water storage containers in both LMSs. Among the three methods (tap, cup, and pour from container) of removing water from the drinking water storage container investigated in this study, pouring water from the container and using of tap that were reported less frequent in the study, remain the best methods that prevent contamination of drinking water storage container. The proportions associated with pour and tap drawing methods were low when compared to that observed in the survey conducted elsewhere^28^. The low use of tap and pour methods observed in this study may be justified by the fact that the respondents could not easily afford a container to which a tap has been adapted at the base. Also, respondents may have found using a cup to remove water from the storage container more convenient than pouring the water from the container. Pouring water out of storage containers was proven by some studies to be effective in preventing contamination of stored water in the home as opposed to the dipping of a cup. In one of the studies^29^, when dipping was employed to draw water from storage containers, an increased risk of drinking water contamination was noted. Another study^28^ discovered that dipping water-drawing utensils in storage containers polluted the water by introducing dirt and dust from the surroundings.

The most stringent hygiene standard is basic, which necessitates the presence of a handwashing station, with soap and water on the premises. In this study, the number of households across both LMSs with limited hygiene facilities was much higher than those with basic hygiene facilities. Although respondents in Amansea LMS were better than those in Ugwuoba LMS in terms of hygiene practice, the discrepancy between households with limited and basic hygiene facilities was wide, and shows that hygiene practice is poor across both LMSs.^30^ also reported was wide discrepancies between the proportions of households having limited hygiene facilities (53.04%) and those having basic hygiene facilities (8.0%). Both the hygiene practices reported in this study and that reported by^31^ are far below the global hygiene practices reported by^32^ who showed that, in 2017, 60% of the world population had basic handwashing facilities with water and soap, whereas 22% had limited handwashing facilities lacking water and/or soap.

The low level of basic handwashing facilities observed in this study could be due to lack of awareness about the importance of hand hygiene. It could also be due to the inability to regularly afford resources such as soap or detergent for handwashing. When hands are not washed properly with clean running water and soap at important periods such as after defecating, before eating, before cooking and so on, many diseases (including diarrhoeal diseases) might spread. Many studies have highlighted the importance of washing hands with soap and water as opposed to using water only in handwashing. In comparing handwashing without soap, one study has shown that handwashing with soap is more efficient at preventing childhood diarrhoea episodes as the practice results in lowering diarrhoea-related mortality and morbidity rates^31^. According to a community-based randomized control experiment involving mothers in Bangladesh^33^, handwashing with a bar of soap was found to be more efficacious in lowering the bacterial load of coliforms and Clostridium perfringens than handwashing with water alone. In the study, a good number of respondents admitted they used soap on the day of and the day before the survey. Similarly, a study by^34^ reported that 80.9% (599/740) of the respondents of his survey washed their hands the previous day and at critical times.

United Nations (UN) Sustainable Development Goal-SDG 6.2 states, “By 2030, achieve access to adequate and equitable sanitation and hygiene for all and end open defecation”. Four years left to reach the year, 2030, the sanitation scenario at Amansea LMS and Ugwuoba LMS is far from meeting this UN target. High proportions of households who still practice open defecation were recorded in both LMSs. The proportions recorded in the study were much higher than that reported elsewhere^35^,^36^. Open defecation has direct consequences as it impedes public health, pollutes water bodies, and affects sanitation. The high rate of open defecation in this study could be due to lack of sufficient resources to build adequate sanitation facilities. Also, these livestock market settlement dwellers believe that their current habitats are temporal and hope that the government gives them more spacious land for their cattle and for them to settle more properly. With this view in mind, it is difficult to build toilets especially when they may no longer be theirs due to possible relocation. Safe faeces disposal is a huge concern, not just for the 2.4 billion people globally who lack access to improved sanitation, but also because of unsanitary faeces collection and disposal, as well as poor handwashing practices.

The most common under 5-year old defecation practice in households within Amansea LMS and Ugwuoba LMS was the use of potty to defecate and defecation outside premises, which are subsequently disposed outside the yard or buried, respectively. These practices have been observed in another country^37^ though at much lower rates. Accordingly, the only safe technique of child faeces management is to properly dispose of child excrement in a toilet or latrine connected to a safe sanitation chain or to assist the child in using a toilet^38^. Other means of disposal (faeces poured or washed into a drain or ditch, shrub, or garbage, buried or left on the ground, and not disposed of) are deemed unsafe. The results of this study show that the majority of households under 5 years old practice unsafe child defecation and child faeces disposal methods. Such practices are a public health threat not just to the children themselves but also to the household members especially the mothers or babysitters and the surrounding communities because, in the event of heavy rainfall, the unsafely disposed of faeces will eventually contaminate water bodies that are used for drinking, irrigation, and other domestic purposes.

In 2017, more than two billion people, roughly a quarter of the world's population did not have access to “sufficient” sanitation services, with 627 million of them using shared toilets rather than a private toilet facility^39^. In this study, it was observed that among those households who used toilet facilities (those not practicing open defecation), private toilet were more common followed by shared and public toilets in both LMSs. This study reveals that the sanitation facilities used by households having toilet facilities across both LMSs were more adequate in the sense that, fewer households with shared sanitation facilities and a majority of households with private sanitation facilities were recorded. A private toilet is preferred to a shared toilet because shared toilets have issues regarding accessibility and cleanliness. It was observed that among those having toilet facilities, a majority of households in both Amansea and Ugwuoba LMSs use sanitation facilities that are improved and basic.

The fact that TCC and FCC were non-detectable in both sachet brands may mean that the disinfection protocol applied by both sachet water companies is adequate making their sachet water suitable for drinking by the Amansea livestock market settlement dwellers as they meet NSDWQ and WHO guidelines for drinking water. Amongst the predominant drinking water sources at Amansea, borehole water recorded significantly higher total and faecal coliform counts. The presence of FC and TC bacteria in the borehole water is still disturbing and may be due to contamination of borehole water by pathogens from nearby waste pits and septic tanks that penetrate the groundwater aquifers and infiltrate deep into the ground. Recent studies by^39^,^40^ also provided similar findings in association with water from boreholes.

One limitation of the current study is that the sanitation indicator only indicate the presence of separate toilets among households without taking into consideration the visitor's aspect. Secondly, though we could generate data related to WASH, it is difficult to assess the level of compliance with regards to environmental cleaning protocols.

## Conclusion

Nearly half of the households investigated in the current study did not have access to improved drinking water sources. Only few of the households treated their drinking water and the borehole water quality did not conform to the WHO guidelines for FCC and TCC in drinking water. Thus, more awareness is needed in both LMSs to highlight the significance of treating water before drinking. Inadequate hygiene practices were recorded in the study as a few households in both LMSs have handwashing facilities in their homes. Even where handwashing facilities were available, fewer people have soap and water available and a few respondents in both LMSs wash their hands after critical points such as after defecating, a common practice in both LMSs. In the current study, the inadequacy of hygiene, and sanitation practices and the failure to treat drinking water amongst a majority of households in Amansea and Ugwuoba LMSs raises a potential concern as these may increase the risk of disease transmission in the area. In-view of the above, we recommend that the Ministry of Health liase with relevant agencies to increase households' access to improved drinking water, hygiene, and sanitation facilities in both LMSs.
